# Supplementary material for: The Autism Spectrum Disorder-Associated Bacterial Metabolite p-Cresol Derails the Neuroimmune Response of Microglial Cells Partially via Reduction of ADAM17 and ADAM10
Source: Int J Mol Sci. 2022 Sep 20;23(19):11013. doi: 10.3390/ijms231911013 (PMC9570133; doi:10.3390/ijms231911013)
Supplement: Supplementary file 1 [file ijms-23-11013-s001.zip › ijms-1867200-supplementary.pdf]

International Journal of *Molecular Sciences*

MDPI

Article

The autism spectrum disorder-associated bacterial metabolite *p*-cresol derails the neuroimmune response of microglial cells partially via reduction of ADAM17 and ADAM10

Yuanpeng Zheng[1], Naika Z. Prince[1], Lucia N. Peralta Marzal[1], Sabbir Ahmed[1], Johan Garssen[1,2], Paula Perez Pardo[1], Aletta D. Kraneveld[1]

[1] Division of Pharmacology, Utrecht Institute for Pharmaceutical Sciences, Faculty of Science, Utrecht University, Utrecht, the Netherlands

[2] Global Centre of Excellence Immunology, Danone-Nutricia research, Utrecht, the Netherlands

* Correspondence: Aletta D. Kraneveld, a.d.kraneveld@uu.nl; Tel: +31-(0)30-2534509

Supplementary materials & methods

1. Isolation of brain tissues and blood collection of *in utero* valproic acid murine model of ASD

Specific pathogen-free BALB/cByJ breeding pairs from Charles River laboratories (Maastricht, the Netherlands) were housed under a 12 h light/dark cycle with free access to food and water. All animal procedures were conducted according to governmental guidelines and approved by the Ethical Committee of Animal Research of Utrecht University, Utrecht, the Netherlands (CCD number AVD108002017826). All females were mated until a vaginal plug was detected, indicated as gestational day 0 (G0). On G11, after neural tube closure, pregnant females were treated subcutaneously with 600 mg/kg valproic acid (VPA, Sigma, Zwijndrecht, the Netherlands, VPA: 100 mg/ml) or phosphate buffered saline (PBS) [1, 2]. The offspring were weaned on postnatal day 21 (P21). On postnatal day 50, male mice were euthanized by decapitation to collect blood and brain.

To assess systemic *p*CS levels, blood was collected in MiniCollect Tube (450533, Greiner BIO-ONE, the Netherlands), then it was centrifugated at 20,000 g, 10 min, 4°C. Next the serum was collected for *p*CS and *p*CG measurements by LC-MS/MS.

To assess ADAM10 and ADAM17 (see: material & methods 3) as well as neuroinflammation (see: materials & methods 5), the following brain regions were isolated and collected: hippocampus, prefrontal cortex, cerebellum and olfactory bulbs, and the rest of brain tissue was also collected. The different brain regions were homogenized at 4 °C using the STET buffer without detergents (50 mM Tris, pH 7.5, 150 mM NaCl, 2 mM EDTA, Proteinase Inhibitor Cocktail (1:200, P8340, Sigma) 5 µM GI254023X (SML0789, Sigma), 10 mM 1,10-Phenanthroline (131377, Sigma)) [3]. After centrifugation at 12,000 g, 15 min, 4°C, the supernatants were collected for ELISA measurements. For immunoblotting, brain tissue was further lysed on ice for 30 min with RIPA buffer (20188, Sigma) containing 1% TritonX-100 detergent, Proteinase Inhibitor Cocktail (1:200), 5 µM GI254023X and 10 mM 1,10-Phenanthroline, next it was centrifugated again at 12,000 g, 15 min, 4°C to collect the supernatant for Western blotting analysis, and tissue debris were discarded.

2. Assessment *p*-cresol metabolites in serum of *in utero* VPA murine model of ASD

*p*CS and *p*CG were purchased from AlsaChim (Illkirch-Graffenstaden, France). d7-*p*-cresyl sulfate (potassium salt) and d7-*p*-cresyl glucuronide were purchased from IsoSciences (Ambler, PA, USA) and Toronto Research Chemicals (North York, Ontario, Canada), respectively. Water (LC-MS grade) and acetonitrile (CAN; HPLC-S grade) were obtained from Biosolve (Valkenswaard, The Netherlands). LC-MS/MS equipment and software for controlling, data recording and processing (Xcalibur version 2.07) were supplied by Thermo Fischer Scientific (San Jose, CA, USA).

Serum samples were processed prior to LC-MS analysis. A 20-µL serum or surrogate matrix (for calibration and quality control) was added to 30 µL of cold (4 °C) ACN containing internal standards d7-*p*CS and d7-*p*CG, and subsequently vortexed and centrifuged for 5 minutes at 10000g. A 35-µL of the supernatant was collected and diluted with 200 µl of ultra-pure water before analysis with an Accela LC system (quaternary pump and autosampler), coupled to a TSQ Quantum Ultra triple quadrupole mass spectrometer with heated electrospray ionization (ESI). A Waters ACQUITY UPLC HSS T3 column (100 mm × 2.1 mm, 1.8 µm particles) combined with an ACQUITY UPLC HSS T3 VanGuard pre-column (5 mm × 2.1 mm, 1.8 µm particles) was used and kept at a temperature of 40 °C.

Supplementary results

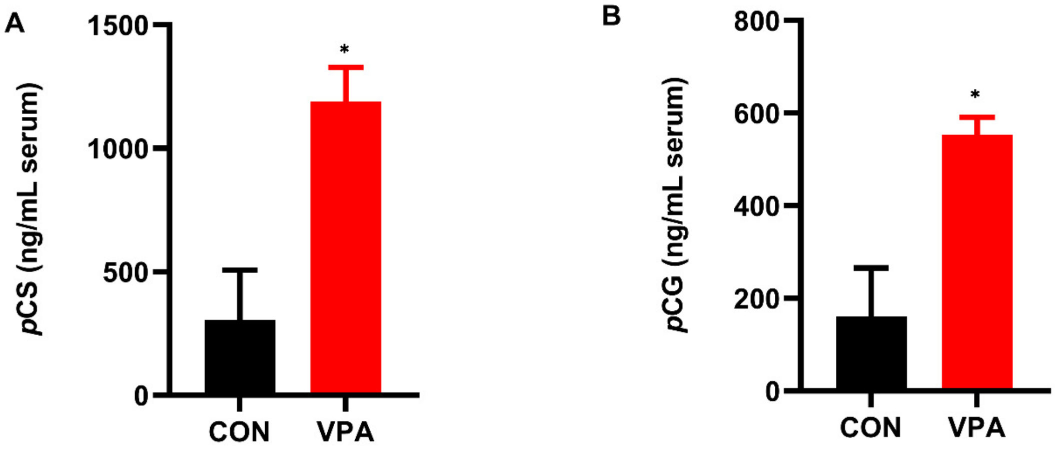

Figure S1. Effects of *in utero* exposure to valproic acid (VPA) on *p*-cresyl sulfate (*p*CS, A) and *p*-cresyl glucuronide (*p*CG, B) levels in serum of male offspring. Results are expressed as mean ± SEM; *$P < 0.05$. n=3 *in utero* VPA-exposed mice; n=6 control mice.

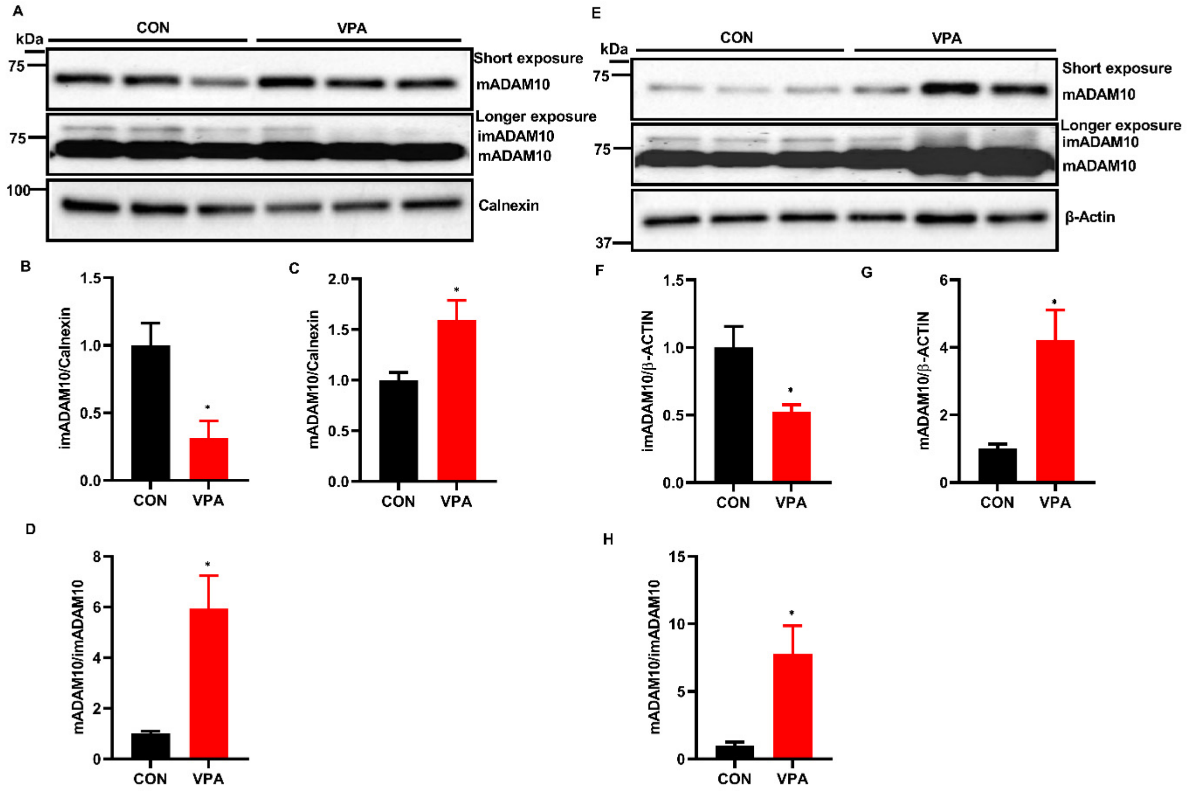

Figure S2. Effects of *in utero* exposure to valproic acid (VPA) on ADAM10 protein expression and maturation efficiency in the brain of male mice. (A) The immunoblots of hippocampal ADAM10. (B, C & D) The quantification results of hippocampal immature ADAM10 (imADAM10), mature ADAM10 (mADAM10) and maturation efficiency (ratio mADAM10/imADAM10). (E) The immunoblots of ADAM10 in other brain regions (excluding hippocampus, olfactory bulb, PFC and cerebellum). (F, G & H) The quantification results of imADAM10, mADAM10 and the maturation efficiency (ratio mDAM10/imADAM10) in other brain regions. Calnexin or β-Actin was used as a loading control. Results were expressed as mean ± SEM. $*P < 0.05$. n=3 VPA-exposed and control mice.

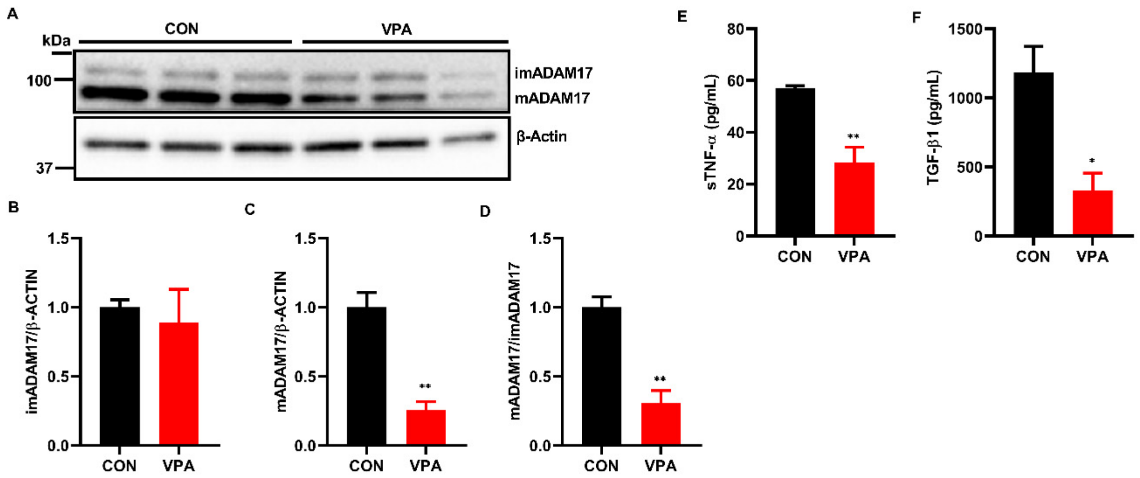

Figure S3. The effects of *in utero* exposure to valproic acid (VPA) on ADAM17 protein expression and levels of soluble TNF-α and TGF-β1 in the hippocampus of male mice. (A) Immunoblots of hippocampal ADAM17. (B, C & D) The quantification results of hippocampal immature ADAM17 (imADAM17), mature ADAM17 (mADAM17) and maturation efficiency (mADAM17/imADAM17 ratio). β-Actin was used as loading control for western blotting. (E & F) The hippocampal soluble TNF-α (sTNF-α) and TGF-β1 levels. Results were expressed as mean ± SEM, n=3. *$P < 0.05$, **$P < 0.01$.

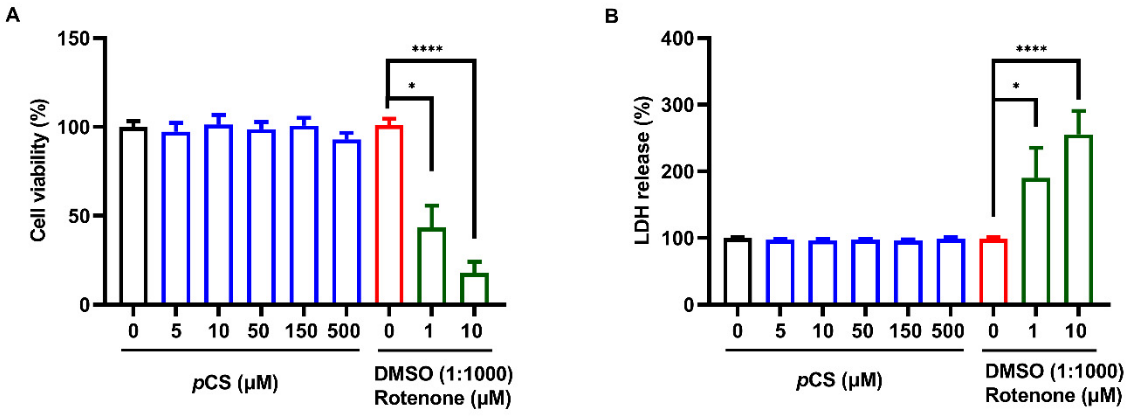

Figure S4. The cell viability measurements with *p*CS treatment in BV2 microglial cells. 5.000 BV2 microglia was seeded into 96-well plate each well overnight, next it was incubated with a concentration range of *p*CS treatments for 24 hours. (A) The cell viability percentage with *p*CS. (B) The LDH release in medium with *p*CS. n=4 from 4 independent experiments. Results were expressed as mean ± SEM. *$*P < 0.05$, $****P < 0.0001$*. Black: cell viability under control condition set at 100%; red: effect of vehicle (DMSO) on cell viability; blue: effect of *p*CS on cell viability; green: effect of rotenone on cell viability.

Reference

1. de Theije, C. G.; Koelink, P. J.; Korte-Bouws, G. A.; Lopes da Silva, S.; Korte, S. M.; Olivier, B.; Garssen, J.; Kraneveld, A. D., Intestinal inflammation in a murine model of autism spectrum disorders. *Brain, behavior, and immunity* **2014,** 37, 240-7.
2. de Theije, C. G.; Wopereis, H.; Ramadan, M.; van Eijndthoven, T.; Lambert, J.; Knol, J.; Garssen, J.; Kraneveld, A. D.; Oozeer, R., Altered gut microbiota and activity in a murine model of autism spectrum disorders. *Brain, behavior, and immunity* **2014,** 37, 197-206.
3. Brummer, T.; Pigoni, M.; Rossello, A.; Wang, H.; Noy, P. J.; Tomlinson, M. G.; Blobel, C. P.; Lichtenthaler, S. F., The metalloprotease ADAM10 (a disintegrin and metalloprotease 10) undergoes rapid, postlysis autocatalytic degradation. *FASEB journal : official publication of the Federation of American Societies for Experimental Biology* **2018,** 32, (7), 3560-3573.
